# Total mesorectal excision after rectal-sparing approach in locally advanced rectal cancer patients after neoadjuvant treatment: a high volume center experience

**DOI:** 10.1177/26317745241231098

**Published:** 2024-06-24

**Authors:** Daniela Rega, Vincenza Granata, Carmela Romano, Roberta Fusco, Alessia Aversano, Vincenzo Ravo, Antonella Petrillo, Biagio Pecori, Elena Di Girolamo, Fabiana Tatangelo, Antonio Avallone, Paolo Delrio

**Affiliations:** Colorectal Surgical Oncology, Department of Abdominal Oncology, Istituto Nazionale Tumori-IRCCS “Fondazione G. Pascale”, Via Semmola 2, Naples 80131, Italy; Radiology Division, Istituto Nazionale Tumori-IRCCS “Fondazione G. Pascale”, Naples, Italy; Experimental Clinical Abdominal Oncology, Department of Abdominal Oncology, Istituto; Nazionale Tumori-IRCCS “Fondazione G. Pascale”, Naples, Italy; Medical Oncology Division, Igea SpA, Napoli, Italy; Colorectal Surgical Oncology, Department of Abdominal Oncology, Istituto Nazionale Tumori-IRCCS “Fondazione G. Pascale”, Naples, Italy; Radiation Therapy, Istituto Nazionale Tumori-IRCCS “Fondazione G. Pascale”, Naples, Italy; Radiology Division, Istituto Nazionale Tumori-IRCCS “Fondazione G. Pascale”, Naples, Italy; Radioprotection and Innovative Technologies, Istituto Nazionale Tumori IRCCS Fondazione; Pascale-IRCCS di Napoli, Naples, Italy; Gastroenterology and Endoscopy Unit, Department of Abdominal Oncology, Istituto; Nazionale Tumori-IRCCS “Fondazione G. Pascale”, Naples, Italy; Pathology and Cytopathology Unit, Department of Support to Cancer Pathways Diagnostics Area, Istituto Nazionale Tumori-IRCCS “Fondazione G. Pascale”, Naples, Italy; Experimental Clinical Abdominal Oncology, Department of Abdominal Oncology, Istituto; Nazionale Tumori-IRCCS “Fondazione G. Pascale”, Naples, Italy; Colorectal Surgical Oncology, Department of Abdominal Oncology, Istituto Nazionale Tumori-IRCCS “Fondazione G. Pascale”, Naples, Italy

**Keywords:** complete clinical response, local recurrence, local regrowth, major clinical response, neoadjuvant treatment, rectal cancer, rectal sparing, salvage surgery

## Abstract

**Background:**

In patient with a complete or near-complete clinical response after neoadjuvant treatment for locally advanced rectal cancer, the organ-sparing approach [watch & wait (W&W) or local excision (LE)] is a possible alternative to major rectal resection. Although, in case of local recurrence or regrowth, after these treatments, a total mesorectal excision (TME) can be operated.

**Method:**

In this retrospective study, we selected 120 patients with locally advanced rectal cancer (LARC) who had a complete or near-complete clinical response after neoadjuvant treatment, from June 2011 to June 2021. Among them, 41 patients were managed by W&W approach, whereas 79 patients were managed by LE. Twenty-three patients underwent salvage TME for an unfavorable histology after LE (11 patients) or a local recurrence/regrowth (seven patients in LE group – five patients in W&W group), with a median follow-up of 42 months.

**Results:**

Following salvage TME, no patients died within 30 days; serious adverse events occurred in four patients; 8 (34.8%) patients had a definitive stoma; 8 (34.8%) patients undergone to major surgery for unfavorable histology after LE – a complete response was confirmed.

**Conclusion:**

Notably active surveillance after rectal sparing allows prompt identifying signs of regrowth or relapse leading to a radical TME. Rectal sparing is a possible strategy for LARC patients although an active surveillance is necessary.

## Introduction

The treatment of rectal cancer needs a multidisciplinary approach performed by a highly specialized multidisciplinary team. Neoadjuvant therapy (NT) – radio or chemoradiotherapy – associated with total mesorectal excision (TME), is the standard of care for locally advanced rectal cancer (LARC) (stage II/III) patients. The goals of a NT are the risk reduction of a local recurrence, tumors downstaging and downsizing, sterilization of perirectal lymph nodes, directed to obtain a better prognosis.^1–3^ TME, even more if preceded by neoadjuvant treatment, is associated with a still measurable postoperative mortality, high rates of morbidity, as genitourinary and sexual dysfunction, long-term functional bowel disturbance and altered fecal continence, stoma complications, and a related negative impact on quality of life (QoL).^4–9^ A complete clinical response (cCR) is described in 20–49% of the patients following NT, and a pathological complete response (pCR) is described in 10–20% of patients.^10–14^ This is associated with favorable long-term patient outcomes compared with those without complete response.^11,15^

In patients with complete or near-complete clinical response, organ-sparing approach – by LE or watch-&-wait (W&W) strategy – could reduce the adverse effects associated with TME^10,16,17^ with a major impact in patient’s QoL, without substantially compromising oncological outcomes.^18–21^

Local recurrence or regrowth occurs in 15–25% of these patients, and distant metastases occur until to 13%.^10,14,20–23^ Therefore, when a rectal-sparing approach is realized, an intensive surveillance of these patients is required and a salvage TME is mandatory for an unfavorable histology after LE or in case of local recurrence or regrowth.

In this retrospective study, we described our experience in rectal-sparing strategy evaluating results and risk in terms of tumor recurrence and evaluated the TME outcome when performed after this approach.

## Materials and methods

From June 2011 to June 2021, we treated 931 patients with LARC by NT. Patients with any T and N+ clinical stage, and/or with circumferential resection margin (CRM) ⩽ 1 mm (by magnetic resonance imaging or endorectal ultrasound for the patients with persistent contraindications to MRI), underwent to radiochemotherapy (50 Gy with concomitant capecitabine at a daily dose of 825 mg/m^2^/12 h); patients with cT2/3, N0, < 5 cm from anal verge and patients facing tumors with enlarged nodes and/or CRM positive who resulted unfit for chemo-radiation, underwent to short course of radiotherapy (25 Gy). The initial stage of patients is reported in Table 1.

**Table 1.** Pre NT stage and neoadjuvant treatments.

Stage	Patients	NT treatments	Notes
I	8	Short course radiotherapy	For all patients the tumor was cT2N0 at <5 cm from the anal verge
II	33	32 pts: short course radiotherapy 1 pt: long course radiochemotherapy	For 32 patients the tumor was cT3N0 with the tumor at <5 cm from the anal verge For one patients the tumor was cT3N0 MCR+ at 8 cm from the anal verge
III	79	30 pts: short course radiotherapy 49 pts: long course radiochemotherapy	30 pts were unfit for long course chemoradiotherapy

NT, Neoadjuvant therapy.

All consecutive patients managed by rectal-sparing strategy, due to a complete or near-complete clinical and radiological response following NT for LARC, from June 2011 to June 2021, were registered in a prospective database. The National Cancer Institute of Naples Ethical Committee board approved the use of data for this retrospective study.

The response evaluation following NT was performed after 8 weeks, from completion of therapies and it included: digital rectal examination, carcinoembryonic antigen test, rigid proctoscopy, whole-body computed tomography (CT) and pelvic MRI.

All cases were rediscussed in a multidisciplinary team meeting (MDT).

The criteria identifying a near complete clinical response were considered^24,25^:

– Minor mucosal irregularity and movable, felt on digital exam
– Small superficial ulceration at proctoscopy (<2 cm)
– Predominant fibrosis (>75%) without lymph node metastasis on MRI assessment

All patients encountered these criteria and agreed to a LE strategy (79 patients) were managed by full-thickness excision.

Clinical complete response was defined by^24,25^:

– The presence of complete tumor regression without lymph node metastasis at MRI evaluation
– White scars or telangiectasia of the mucosa, the absence of mass, ulceration, or stenosis at proctoscopy

All patients encountered these criteria and agreed to W&W (41 patients) were approached by W&W strategy.

Patients with poor response after NT, lymph node involvement, or metastases at restaging imaging after NT were excluded.

**Figure 1.**
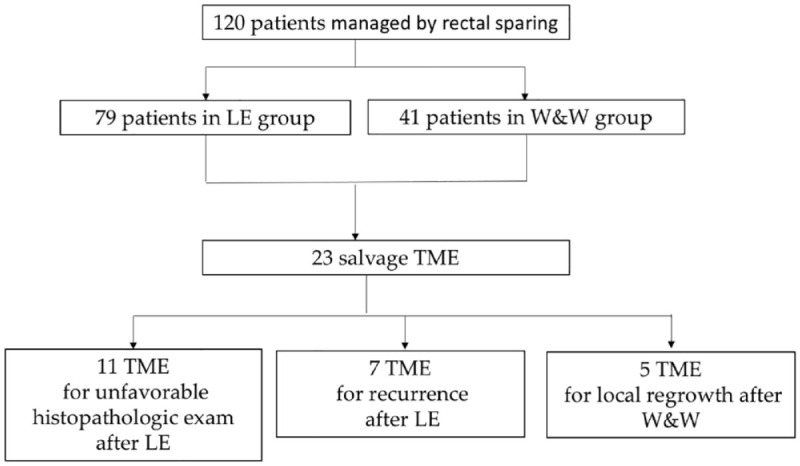
Flowchart of the study.

Salvage TME was performed in (Figure 1):

– Patients with histopathologic evidence of ypT ⩾ 2 ± R ⩾ 1, after LE
– Patients with local recurrence or regrowth during follow-up

TNM classification was utilized^26^ to stage tumors before and after NT.

Pathologic assessment of tumor regression was carried out according to the Mandard’s classification.^27^

Postoperative morbidity was assessed according to the Clavien–Dindo classification.^28^

### Follow-up

Our follow-up strategy included clinical examination, carcinoembryonic antigen test, and rigid proctoscopy in every outpatient visit, every 3 months during the first 2 years, then every 6 months in the following 3 years, and then every 12 months in the following 5 years.

The radiological follow-up included MRI every 6 months and whole-body CT every year for distant metastases.

Colonoscopy was performed after 1 year and then every 2 years.

Local regrowth and local recurrence were defined as any reappearance of tumor at the original tumor location or nodal recurrence in the pelvis.

Distant metastases were defined as the presence of metastatic disease, as identified by CT.

### Statistical analysis

Yates’ chi square test was employed to analyze differences of categorical variable prevalence, whereas the nonparametric Kruskal–Wallis test was used to test for statistically significant differences between the median values of the continuous variables. Disease-free survival rate as the percentage of treated patients who still have no evidence of disease at a certain period of time after treatment. Overall survival rate was calculated as the proportion of patients still alive at a given period of time after treatment.

A *p-*value < 0.05 was considered as statistically significant.

Statistical analysis was obtained by means of the Statistic Toolbox of MATLAB (The MathWorks, Inc., Natick, MA, USA).

## Results

During a frame time of 10 years, a total of 120 patients (Figure 1) treated by NT for LARC had a complete or near-complete clinical response at restaging.

In total, 79 patients underwent LE (LE group), and 41 patients underwent W&W strategy (W&W group).

Study group included 35 females and 85 males. The median age was 67 (range 28–87) years, lesion distance from the anal verge (av) was 5.7 (range 4–12) cm (Table 2). The median follow-up was 42 (range 12–138) months.

**Table 2.** Clinical and demographic characteristics of patients.

	LE	W&W
Patients number	79	41
Gender (male/female)	57/22	28/13
Median age (range) years	67 (28–84)	67 (46–87)
Median distance from the av. (range) cm	5.5 (4–10)	6.9 (4–12)
TME	18/79 (22.8%)	5/79 (12.2%)
ypT ⩾ 2 ± R ⩾ 1	11/18	
Local recurrence/regrowth during follow-up	7/18	5/5

LE, local excision; TME, total mesorectal excision; W&W, watch & wait.

Salvage TME after the rectal-sparing approach was performed in 23 patients (Table 2) with the following indications:

– Eleven patients with evidence of ypT ⩾ 2 ± R ⩾ 1, at histopathologic exam after LE.
– Seven patients with local recurrence after LE during follow-up.
– Five patients with local regrowth after W&W strategy during follow-up.

Of those operated on 15 patients (65.2%) were colostomy-free.

Five exits were recorded in the LE group in the 12–68 month range. No exit was recorded in the W&W group. The 5-year overall survival rate was 97.5%.

In Figure 1, we outline the flowchart of the study.

### LE group

LE group (79 patients) included 22 females and 57 males. The median age was 67 (range 28–84) years, lesion distance from the anal verge was 5.5 (4–10)cm.

LE was performed either with the traditional transanal excision (TAE) or by a minimally invasive approach by transanal endoscopic microsurgery (TEM) or transanal minimally invasive surgery (TAMIS). Median operative time for full thickness local excision (LE) of residual tumor has been 57 (range 10–145) min; hospital stay was 3 (range 1–16) days. Serious intraoperative complications were peritoneal reflection opening (two patients – 2.5%), treated by transanal suture; in only three patients, there was a nonhealing rectal wound with a mild discomfort resulted into at most 3 weeks; no serious postoperative adverse events (Clavien–Dindo ⩾ 3) occurred (Table 3).

**Table 3.** Results about LE group.

Local excision group – 79 patients	
TAE	32 procedures
TEM	2 procedures
TAMIS	45 procedures
Median operative time	57 min (range 10–45)
Hospital stay	3 days (range 1–6)
Intraoperative complications	2 patients
Histological exam	
ypT0 TRG1	33 (41.8%) patients
ypT1 TRG2 R0	21 (26.6%) patients
ypT1 R1	1 (1.2%) patient
ypT2	24 (30.4%) patients

LE, local excision; TAE, transanal excision; TAMIS, transanal minimally invasive surgery; TEM, transanal endoscopic microsurgery.

Histology confirmed 33 (41.8%) patients with ypT0 TRG1 and 21 (26.6%) patients with ypT1 R0 TRG2 (Table 3).

In 25 (31.6%) patients, histology showed ypT ⩾ 2 and/or R ⩾ 1 (1 patient with ypT1 R1, 24 patients with ypT2). Out of these, radical surgical salvage was performed on 11 patients; 14 patients did not undergo to major surgery: eight patients were considered unfit for a completion surgery and six patients refused major surgery. We included these patients in the results of the follow-up.

TME for unfavorable histopathologic exam after LE.

We performed TME in 11 patients with unfavorable histopathologic results following LE: ypT ⩾ 2 and/or R ⩾ 1 (Table 4).

**Table 4.** TME for unfavorable histopathology after LE.

ypT ⩾ 2 and/or R ⩾ 1 after LE	11 patients underwent to TME
LAR with colorectal anastomoses	2 (18.2%)
LAR with coloanal anastomoses (pull through)	6 (54.5%)
Abdominoperineal rectal resection	3 (27.3%)
Median operative time	166 min (105–240)
Hospital stay	10 days (7–17)
Postoperative complications	1 patient (9%)
Histopathologic exam	
ypTxN0 TRG1	8 (72.7%) patients
ypT2N0	3 (27.3%) patients

LAR, low anterior rectal; LE, local excision; TME, total mesorectal excision.

Two (18.2%) patients underwent low anterior rectal (LAR) resection with colorectal anastomosis, 6 (54.5%) patients underwent LAR resection with coloanal anastomosis (pull through), and 3 (27.3%) patients underwent abdominoperineal rectal resection.

Median operative time was 166 (range 105–240) min and median hospital stay was 10 (range 7–17) days.

The major postoperative complication was anastomotic dehiscence (one patient) treated with conservative approach.

Final histology in this group of patients evidenced absence of tumor (ypTxN0 TRG1) in 8 (72.7%) and ypT2N0 in 3 (27.3%) patients. Mesorectal excision was considered complete in all patients. No patients had unclear margins of resection.

After a median follow-up period of 42 (2–137) months, no patients had local recurrence, and distant metastases were detected in one patient, 32 months after salvage surgery and treated with chemotherapy.

Completion TME for recurrence after LE.

A total of 68 patients were treated by exclusive LE. After a median follow-up period of 48 (range 28–62) months, local recurrence occurred in 7 (10.3%) patients.

In three patients, the recurrence appeared within 12 months, in four patients within 24 months. Distant metastases were detected in 5 (7.3%) patients. The disease-free survival at 48 months was 82.35%.

All patients with local recurrence underwent completion TME. Two patients underwent LAR resection with colorectal anastomoses, four patients underwent abdominoperineal rectal resection, and one patient underwent low anterior rectal resection with colostomy (Table 5).

**Table 5.** TME for recurrence after LE.

Completion TME for recurrence after LE	Seven patients underwent to TME
LAR with colorectal anastomoses	2 (28.6%)
Abdominoperineal rectal resection	4 (57.1%)
LAR with colostomy	1 (14.3%)
Median operative time	172 min (80–210)
Hospital stay	14 days (3–24)
Postoperative complications	2 patients (28.6%)
Histopathologic exam	
ypT1N0	Three patients
ypT2N0	One patient
ypT3N0	One patient
ypT4N0	One patient
ypT0N0V1 TRG1	One patient

LAR, low anterior rectal; LE, local excision; TME, total mesorectal excision.

Median operative time was 172 (range 80–210) min, and hospital stay was 14 (3–24) days.

The major complications were: postoperative anastomotic dehiscence (one patient) treated with conservative approach, and a severe heart failure in one patient.

Final histological exam evidenced ypT1N0 in three patients, ypT2N0 in one patient, ypT3N0 in one patient, ypT4N0 in one patient, and ypT0N0V1 in one patient.

Mesorectal excision was considered curative in all patients. In fact, no patients had R1 margins of resection.

After a successive median follow-up period of 42 (16–137) months, no patients had local recurrence or distant metastases.

### W&W group

A total of 41 patients were approached by W&W strategy. This group included 13 females and 28 males. The median age was 67 (range 46–87) years, lesion distance from the anal verge was 6.9 (4–12) cm. After a median follow-up period of 39 (range 12–96) months, local regrowth occurred in 6 (14.6%) patients. Out of them, five patients underwent salvage TME; one patient refused surgery. The disease-free survival at 48 months was 85.37%.

The regrowth appeared in two patients within 12 months, in one patient within 24 months, in two patients within 3 years, and in one patient within 5 years.

In four patients, anterior rectal resection with intestinal reconstruction was performed; one patient underwent abdominoperineal rectal resection (Table 6).

**Table 6.** TME for regrowth after W&W strategy.

Salvage TME for regrowth after W&W	Five patients
LAR with colorectal anastomoses	4 (80%) patients
Abdominoperineal rectal resection	1 (20%) patient
Median operative time	135 min (95–160)
Hospital stay	12 days (3–30)
Histopathologic exam	
ypT1N0	Two patients
ypT2N0	Two patients
ypT3N1V1	One patient

LAR, low anterior rectal; LE, local excision; TME, total mesorectal excision; W&W, watch & wait.

Median operative time was 135 (range 95–160) min, and hospital stay was 12 (3–30) days. A major complication occurred in one patient (urethral leak, treated by urethral surgical reconstruction).

Final histological exam evidenced ypT1N0 in two patients, ypT2N0 in two patients, and ypT3N1V1 in one patient (this last patient underwent adjuvant therapy).

After a successive median follow-up period of 35 (2–93) months, no patients had local recurrence or distant metastases.

Three patients without local regrowth had distant metastases (after 13, 12, and 9 months) and underwent chemotherapy, surgery, and liver metastases ablation treatment, respectively.

No statistically significant difference in local recurrence/regrowth rate and distant metastasis rate were observed between the patients of LE group compared to the patients of W&W group (*p* value 0.06). A difference, although not statistically significant was reported in TME rate after LE compared to TME rate after W&W (22.8% *versus* 12.2% – *p* value 0.24).

Then, a completion TME surgery was required in 19.2% of cases managed with rectal-sparing approaches, describing a rate 13% of postoperative complications and a rate 65.2% of colostomy-free patients; no local recurrence following salvage surgery and no oncological difference in outcome between primary or late TME occurred.

## Discussion

In recent years, the organ-sparing approach in patients with complete or near-complete response to NT is becoming an alternative treatment to TME. In patients with local recurrence or regrowth or unfavorable histology after organ sparing, the major surgery is still considered a standard of care. The MDT choice toward a rectal-sparing approach is guided by clinical and MRI assessment which helped to predict the pathological response to neoadjuvant treatment.^29–34^ As in many guidelines, an organ-sparing approach has to be considered only within clinical trials and in centers with experienced multidisciplinary teams. Local recurrence/regrowth rates within 2 years following rectal sparing are described in a range from 7 to 23%.^10,14,35,36^

However, there is an open issue of whether the TME protracted interval from NT could cause excessive fibrosis that may lead to increased surgical complexity and postoperative morbidity. Although the optimal timing is still undetermined, few studies have assessed this issue.^33–35^ The high rate of postoperative complications and the high rate of local recurrence related to TME following a compromised mesorectum from LE is one of the crucial point of the rectal sparing’s failure.^37–39^

The aim of the present study was to report the outcomes of TME after organ-sparing approaches to evaluate oncological safety and surgical feasibility.

In our experience, 18 patients underwent a complete TME followed by a local excision and 5 patients followed the wait and watch strategy.

Among patients who agreed to undergo the complete TME surgery for unfavorable histopathology after LE, only 3 of 11 patients showed residual cancer at their final histopathology evaluation. The clinical implication is relevant since eight patients underwent an anterior rectal resection with the absence of residual cancer at the final histopathology, also placing a reflection on the real need, in some cases, to proceed with salvage TME.

In our study, seven patients (10.3%) showed recurrence after LE, requiring a subsequent completion TME. This seems to be in line with other authors who describe a recurrence rate of 5–15%.^40,41^ Other authors^42^ describe different recurrence rate (22%) following rectal sparing. These very broad differences regarding recurrence rates suggest that a precise and communal management for rectal sparing following NT does not yet exist.

All recurring tumors were salvageable by TME. The mean time to the recurrence following LE was 13 months (range 8–21), with a higher rate of local recurrences within 2 years. High rates (42.8%) of local recurrence have been found in patients with ypT2 following LE that refused early salvage surgery.

In our experience, five patients showed regrowth after the W&W approach. The mean time to the local regrowth was 22 months (range 7–56 months) with a higher rate (40%) within the first year; all tumors were salvageable by TME. Two patients (40%) reported an ypT1N0 residual cancer at final histopathology, opening the prospective to evaluate LE for the regrowth too.

We described a postoperative complications rate of 16.6% considering only TME following local excision. This seems to be lower than results reported from other authors who described an increase of morbidity and a worsen quality of the mesorectal plane.^37–39^ This result in our clinical records can be related to the absence of a standard colorectal anastomosis; in six patients, we performed a coloanal pull through anastomosis, and in eight patients we performed a permanent colostomy. However, in our series, all patients had a good quality of the mesorectal plane. Colostomy-free rate was 72.7%.

Regarding the need for a permanent colostomy, it is difficult to establish as this depends on the previous surgery or the height of the primary tumor. In our experience, for two patients the lesion was on the anorectal ring (one of these patients was 80 years old), and in another case, the lesion was 3 cm from the anorectal ring, in a patient with a disabling neuropathic pathology. These pre-existing conditions also guided our surgical decision for a permanent colostomy.

The organ preservation rate is 80.8%, and it is similar to the oldest datasets,^43,44^ despite geographic and patient heterogeneity. Other data series report a higher organ preservation rate.^45,46^ The reasons for this are undoubtedly multifactorial, including selection bias, the cohort volume, age differences, maturity of follow-up, and institutional differences.

Therefore, in our experience, after a median follow-up period of 42 months after salvage TME, no patients had local recurrence. This finding is likely related to tumor stage, being a baseline low-stage patient cohort, and indicating that delayed surgery may not compromise the local control in these previously selected patients.

## Conclusion

In conclusion, in our study, a completion TME surgery was required in 19.2% of cases managed with rectal-sparing approaches, describing a rate 13% of postoperative complications and a rate 65.2% of colostomy-free patients, no local recurrence following salvage surgery and no oncological difference in outcome between primary or late TME occurred.

Our data suggest that patients with an accurate evaluation of complete or near-complete response and then treated with the organ-sparing strategy have no oncological disadvantage neither in case of recurrence, and the surgical and oncological outcomes seem to be comparable to those described for patients who underwent to primary TME surgery, with a complete or near-complete pathologic response.

We support as the salvage TME does not invalidate the oncological outcomes; the prognosis for rectal cancer remain relate with the stage of the tumor, following NT.

In this scenario, it is clear as an active follow-up is necessary.

## Declarations

**Ethics approval and consent to participate:** The study was conducted according to the guidelines of the Declaration of Helsinki, and approved by the Institutional Review Board (“Fondazione Giovanni Pascale” Istituto di Ricovero e Cura a Carattere Scientifico, Via Mariano Semmola – 80131 Napoli (protocol code 551, date of approval 24 July 2019). Informed consent was obtained from all subjects involved in the study.

**Consent for publication:** Not applicable.

**Availability of data and materials:** Data available in a publicly accessible repository.

## References

[1] Glynne-Jones R Wyrwicz L Tiret E , et al.; ESMO Guidelines Committee. Rectal cancer: ESMO clinical practice guidelines for diagnosis, treatment and follow-up. Ann Oncol 2017; 28(Suppl. 4): iv22–iv40. 10.1093/annonc/mdx224 28881920

[bibr2-26317745241231098] Benson AB Venook AP Al-Hawary MM , et al. NCCN guidelines insights: rectal cancer, version 6.2020. J Natl Compr Canc Netw 2020; 18: 806–815. 32634771 10.6004/jnccn.2020.0032

[3] Scala D Niglio A Pace U , et al. Laparoscopic intersphincteric resection: indications and results. Updates Surg 2016; 68: 85–91. 27022927 10.1007/s13304-016-0351-6

[4] Juul T Ahlberg M Biondo S , et al. Low anterior resection syndrome and quality of life: an international multicenter study. Dis Colon Rectum 2014; 57: 585–591. 24819098 10.1097/DCR.0000000000000116

[bibr5-26317745241231098] Emmertsen KJ Laurberg S . Low anterior resection syndrome score: development and validation of a symptom-based scoring system for bowel dysfunction after low anterior resection for rectal cancer. Ann Surg 2012; 255: 922–928. 22504191 10.1097/SLA.0b013e31824f1c21

[bibr6-26317745241231098] Gollins S Moran B Adams R , et al. Association of coloproctology of Great Britain & Ireland (ACPGBI): guidelines for the Management of cancer of the colon, rectum and anus (2017) - multidisciplinary management. Colorectal Dis 2017; 19(Suppl. 1): 37–66. 10.1111/codi.13705 28632307

[bibr7-26317745241231098] Wiltink LM Marijnen CAM Meershoek-Klein Kranenbarg E , et al. A comprehensive longitudinal overview of health-related quality of life and symptoms after treatment for rectal cancer in the TME trial. Acta Oncol 2016; 55: 502–508. 26406287 10.3109/0284186X.2015.1088171

[bibr8-26317745241231098] Pucciarelli S Del BP Pace U , et al. Multicentre randomized clinical trial of colonic J pouch or straight stapled colorectal reconstruction after low anterior resection for rectal cancer. Br J Surg 2019; 106: 1147–1155. 31233220 10.1002/bjs.11222

[9] Gavaruzzi T Pace U Giandomenico F , et al. Colonic J-Pouch or straight colorectal reconstruction after low anterior resection for rectal cancer: impact on quality of life and bowel function: a multicenter prospective randomized study. Dis Colon Rectum 2020; 63: 1511–1523. 33044292 10.1097/DCR.0000000000001745

[10] Rega D Granata V Romano C , et al. Watch and wait approach for rectal cancer following neoadjuvant treatment: the experience of a high volume cancer center. Diagnostics (Basel) 2021; 11: 1507. 34441441 10.3390/diagnostics11081507 PMC8394713

[bibr11-26317745241231098] Rega D Pecori B Scala D , et al. Evaluation of tumor response after short-course radiotherapy and delayed surgery for rectal cancer. PLoS One 2016; 11: e0160732. 10.1371/journal.pone.0160732 PMC4993446 27548058

[bibr12-26317745241231098] Glynne-Jones R Hughes R . Complete response after chemoradiotherapy in rectal cancer (Watch-and-Wait): have we cracked the code? Clin Oncol (R Coll Radiol) 2016; 28: 152–160. 26625960 10.1016/j.clon.2015.10.011

[bibr13-26317745241231098] Rega D Pace U Niglio A , et al. TAMIS for rectal tumors: advancements of a new approach. Updates Surg 2016; 68: 93–97. 27052544 10.1007/s13304-016-0362-3

[14] Marchegiani F Palatucci V Capelli G , et al. Rectal sparing approach after neoadjuvant therapy in patients with rectal cancer: the preliminary results of the ReSARCh trial. Ann Surg Oncol 2022; 29: 1880–1889. 34855063 10.1245/s10434-021-11121-8

[15] Al-Sukhni E Attwood K Mattson DM , et al. Predictors of pathologic complete response following neoadjuvant chemoradiotherapy for rectal cancer. Ann Surg Oncol 2016; 23: 1177–1186. 26668083 10.1245/s10434-015-5017-y PMC5295136

[16] Stijns RCH de Graaf EJR Punt CJA , et al.; CARTS Study Group. Long-term oncological and functional outcomes of chemoradiotherapy followed by organ-sparing transanal endoscopic microsurgery for distal rectal cancer: the CARTS study. JAMA Surg 2019; 154: 47–54. 30304338 10.1001/jamasurg.2018.3752 PMC6439861

[17] Hahnloser D Cantero R Salgado G , et al. Transanal minimal invasive surgery for rectal lesions: should the defect be closed? Colorectal Dis 2015; 17: 397–402. 25512176 10.1111/codi.12866

[18] Castaldo ET Parikh AA Wright Pinson C , et al. Merchant Improvement of survival with response to neoadjuvant radiation therapy for rectal cancer Arch Surg 2009; 144: 129–134; discussion 134–135. 19221323 10.1001/archsurg.2008.549

[bibr19-26317745241231098] Capirci C Valentini V Cionini L , et al. Prognostic value of pathologic complete response after neoadjuvant therapy in locally advanced rectal cancer: long-term analysis of 566 ypCR patients Int J Radiat Oncol Biol Phys 2008; 72: 99–107. 18407433 10.1016/j.ijrobp.2007.12.019

[bibr20-26317745241231098] Habr-Gama A Perez RO Nadalin W , et al. Operative versus nonoperative treatment for stage 0 distal rectal cancer following chemoradiation therapy: long-term results. Ann Surg 2004; 240: 711–717; discussion 717–718. 15383798 10.1097/01.sla.0000141194.27992.32 PMC1356472

[bibr21-26317745241231098] Marks John H Valsdottir EB DeNittis A , et al. Transanal endoscopic microsurgery for the treatment of rectal cancer: comparison of wound complication rates with and without neoadjuvant radiation therapy. Surg Endosc 2009; 23: 1081–1087. 19263164 10.1007/s00464-009-0326-5

[bibr22-26317745241231098] Dattani M Heald RJ Goussous G , et al. Oncological and survival outcomes in watch and wait patients with a clinical complete response after neoadjuvant chemoradiotherapy for rectal cancer: a systematic review and pooled analysis. Ann Surg 2018; 268: 955–967. 29746338 10.1097/SLA.0000000000002761

[23] van der Valk MJM Hilling DE Bastiaannet E , et al. Long-term outcomes of clinical complete responders after neoadjuvant treatment for rectal cancer in the International Watch & Wait Database (IWWD): an international multicentre registry study. Lancet 2018; 391: 2537–2545. 29976470 10.1016/S0140-6736(18)31078-X

[24] Hupkens BJP Maas M Martens MH , et al. Organ preservation in rectal cancer after chemoradiation: should we extend the observation period in patients with a clinical near-complete response? Ann Surg Oncol 2018: 25; 197–203. 29134378 10.1245/s10434-017-6213-8

[25] Chiloiro G Meldolesi E Giraffa M , et al. Could the conservative approach be considered safe in the treatment of locally advanced rectal cancer in case of a clinical near-complete or complete response? a retrospective analysis. Clin Transl Radiat Oncol 2021: 28: 1–9. 33732909 10.1016/j.ctro.2021.02.009 PMC7937531

[26] www.uicc.org (accessed 1 June 2021).

[27] Mandard AM Dalibard F Mandard JC , et al. Pathologic assessment of tumor regres-sion after preoperative chemoradiotherapy of esophageal carcinoma: clinicopathologic correlations. Cancer 1994; 73, 2680–2686. 8194005 10.1002/1097-0142(19940601)73:11<2680::aid-cncr2820731105>3.0.co;2-c

[28] Dindo D Demartines N Clavien PA . Classification of surgical complications: a new proposal with evaluation in a cohort of 6336 patients and results of a survey. Ann Surg 2004; 240: 205–213. 15273542 10.1097/01.sla.0000133083.54934.ae PMC1360123

[29] Fusco R Granata V Sansone M , et al. Validation of the standardized index of shape tool to analyze DCE-MRI data in the assessment of neo-adjuvant therapy in locally advanced rectal cancer. Radiol Med 2021; 126: 1044–1054. 34041663 10.1007/s11547-021-01369-1

[bibr30-26317745241231098] Fusco R Granata V Rega D , et al. Morphological and functional features prognostic factor of magnetic resonance imaging in locally advanced rectal cancer. Acta Radiol 2019; 60: 815–825. 30286607 10.1177/0284185118803783

[bibr31-26317745241231098] Petrillo A Fusco R Granata V , et al. Assessing response to neo-adjuvant therapy in locally advanced rectal cancer using intra-voxel incoherent motion modelling by DWI data and standardized index of shape from DCE-MRI. Ther Adv Med Oncol 2018; 10: 1758835918809875. 30479672 10.1177/1758835918809875 PMC6243411

[bibr32-26317745241231098] Petrillo A Fusco R Granata V , et al. MR imaging perfusion and diffusion analysis to assess preoperative short course radiotherapy response in locally advanced rectal cancer: standardized Index of Shape by DCE-MRI and intravoxel incoherent motion-derived parameters by DW-MRI. Med Oncol 2017; 34: 198. 29151142 10.1007/s12032-017-1059-2

[bibr33-26317745241231098] Fischer J Eglinton TW Richards SJ , et al. Predicting pathological response to chemoradiotherapy for rectal cancer: a systematic review. Expert Rev Anticancer Ther 2021; 21: 489–500. 33356679 10.1080/14737140.2021.1868992

[bibr34-26317745241231098] Cusumano D Meijer G Lenkowicz J , et al. A field strength independent MR radiomics model to predict pathological complete response in locally advanced rectal cancer. Radiol Med 2021; 126: 421–429. 32833198 10.1007/s11547-020-01266-z PMC7937600

[35] Dattani M Heald RJ Goussous G , et al. Oncological and survival outcomes in watch and wait patients with a clinical complete response after neoadjuvant chemoradiotherapy for rectal cancer: a systematic review and pooled analysis. Ann Surg 2018; 268: 955–967. 29746338 10.1097/SLA.0000000000002761

[36] Dossa F Chesney TR Acuna SA , et al. A watch-and-wait approach for locally advanced rectal cancer after a clinical complete response following neoadjuvant chemoradiation: a systematic review and meta-analysis. Lancet Gastroenterol Hepatol 2017; 2: 501–513. 28479372 10.1016/S2468-1253(17)30074-2

[37] Serra-Aracil X Saldaña AG Mora-Lopez LL , et al. Completion surgery in unfavorable rectal cancer after transanal endoscopic microsurgery: does it achieve satisfactory sphincter preservation, quality of total mesorectal excision specimen, and long-term oncological outcomes? Dis Colon Rectum 2021; 64: 200–208. 33315715 10.1097/DCR.0000000000001730

[bibr38-26317745241231098] Coton C Lefevre JH Debove C , et al. Does transanal local resection increase morbidity for subsequent total mesorectal excision for early rectal cancer? Colorectal Dis 2019; 21: 15–22. 30300969 10.1111/codi.14445

[39] Eid Y Alves A Lubrano J , et al. Does previous transanal excision for early rectal cancer impair surgical outcomes and pathologic findings of completion total mesorectal excision? Results of a systematic review of the literature. J Visc Surg 2018; 155: 445–452. 29657063 10.1016/j.jviscsurg.2018.03.008

[40] van Oostendorp SE Smits LJH Vroom Y , et al. Local recurrence after local excision of early rectal cancer: a meta-analysis of completion TME, adjuvant (chemo)radiation, or no additional treatment. Br J Surg 2020; 107: 1719–1730. 32936943 10.1002/bjs.12040 PMC7692925

[41] Jones HJS Cunningham C Nicholson GA , et al. Outcomes following completion and salvage surgery for early rectal cancer: a systematic review. Eur J Surg Oncol 2018; 44: 15–23. 29174708 10.1016/j.ejso.2017.10.212

[42] Wawok P Polkowski W Richter P , et al. Polish Colorectal Cancer Study Group Preoperative radiotherapy and local excision of rectal cancer: long-term results of a randomised study Radiother Oncol 2018; 127: 396–403. 29680321 10.1016/j.radonc.2018.04.004

[43] Joshua Smith J Strombom P Chow OS , et al. Assessment of a watch-and-wait strategy for rectal cancer in patients with a complete response after neoadjuvant therapy. JAMA Oncol 2019; 5: e185896. 10.1001/jamaoncol.2018.5896 PMC6459120 30629084

[44] Habr-Gama A Gama-Rodrigues J Julião GPS , et al. Local recurrence after complete clinical response and watch and wait in rectal cancer after neoadjuvant chemoradiation: impact of salvage therapy on local disease control. Int J Radiat Oncol Biol Phys 2014; 88: 822–828. 24495589 10.1016/j.ijrobp.2013.12.012

[45] Martens MH Maas M Heijnen LA , et al. Long-term outcome of an organ preservation program after neoadjuvant treatment for rectal cancer. J Natl Cancer Inst 2016; 108: djw171. 10.1093/jnci/djw171 27509881

[46] Renehan AG Malcomson L Emsley R , et al. Watch-and-wait approach versus surgical resection after chemoradiotherapy for patients with rectal cancer (the OnCoRe project): a propensity-score matched cohort analysis. Lancet Oncol 2016; 17: 174–183. 26705854 10.1016/S1470-2045(15)00467-2

